# Healthcare use and sexually transmitted infections treatment-seeking: a mixed methods cross-sectional survey among hard-to-reach fishing communities of Lake Victoria, Uganda

**DOI:** 10.11604/pamj.2024.48.134.27244

**Published:** 2024-07-24

**Authors:** Ali Ssetaala, Sabrina Welsh, Teddy Nakaweesa, Mathias Wambuzi, Gertrude Nanyonjo, Annet Nanvubya, Juliet Mpendo, Annet Nalutaaya, Julius Ssempiira, Leslie Nielsen, Pat Fast, Matt Price, Noah Kiwanuka

**Affiliations:** 1 Uganda Virus Research Institute (UVRI), International AIDS Vaccine Initiative (IAVI), HIV Vaccine Program Limited, Entebbe, Uganda,; 2 Human Vaccines Project, New York, United States of America,; 3 Uganda Tuberculosis Implementation Research Consortium, Kampala, Uganda,; 4 Department of Epidemiology and Biostatistics, School of Public Health, Makerere University, Kampala, Uganda,; 5 Partnership for Research on Ebola Virus in Liberia (PREVAIL), Monrovia, Liberia,; 6 International AIDS Vaccine Initiative (IAVI), New York, United States of America,; 7 Department of Epidemiology and Biostatistics, University of California at San Francisco, San Francisco, California, United States of America

**Keywords:** Healthcare, treatment, seeking, sexually transmitted infections, barriers

## Abstract

**Introduction:**

Uganda´s fishing communities experience a high burden of sexually transmitted infections (STIs) including human immunodeficiency virus (HIV), with limited access to healthcare. Knowledge on healthcare use and treatment seeking will help identify unmet needs and facilitate appropriate allocation of resources.

**Methods:**

between 2014-2015, a mixed methods cross-sectional survey was conducted in four fishing communities on Lake Victoria, Uganda, as part of preparedness for HIV trials. The goal was to understand health problems (having any illness, medical condition, or injury in the past 12 months), perceptions of healthcare, health services use, and factors associated with seeking STI care. Data were collected from participants aged 13-49 years; quantitatively using a structured questionnaire and qualitatively via focus group discussions (FGDs) and key informant interviews (KIIs). Information covered recent health problems, health services use, and healthcare perceptions. Multivariable logistic regression modeling was used to determine factors associated with seeking care for STIs.

**Results:**

participants´ median (interquartile range) age was 29 (23-35) years, more than half (51.9%, 763/1,469), were females, and the majority (60.4%, 888/1,469) had up to seven years of formal education. Most participants reported having had health problems (76%, 1,117/1,469). The most frequently reported health issues were STI symptoms (52.6%, 587/1,117). Lack of health services was mentioned as one of the reasons for not seeking care during the FDGs and KIIs. Adolescents, 13-19 were less likely to seek care for STIs symptoms than adults of 20 or more years (aOR= 0.5 (95% CI 0.3-0.9)). Females were more likely to seek STI treatment (aOR= 1.4 (95% CI 1.0-2.1)), as were participants who worked mainly in bars, restaurants or lodges (aOR= 2.0 (95% CI 1.1-3.6)).

**Conclusion:**

in these communities, adolescents have low treatment seeking for STIs symptoms.

## Introduction

Fishing communities (FCs) in Uganda experience a high burden of HIV and sexually transmitted infections (STIs) and may have limited access to healthcare, especially in hard-to-reach communities where health facilities and resources are scarce [1-3]. Access to health services and their utilization are important for the improvement of health outcomes like maternal mortality, child mortality, and reduction in disease burden. Understanding access and utilization of health services helps design better health interventions and improves healthcare coverage, especially in hard-to-reach rural communities like the FCs [4]. Residents of FCs are getting involved more frequently in health research including HIV prevention clinical trials. Identifying medical conditions affecting these communities and how they use health care services is important to understanding the baseline comorbidities and health needs in the FCs.

Fishing communities may experience challenges that affect residents´ and access to and use of health care, leading to a high burden of diseases like STIs and HIV. A variety of factors can influence how individuals seek and use health care services, including but not limited to cultural beliefs, distance to health service centers, availability of transport means, availability of health services and care providers, affordability of care, quality of care, and health system policies [5]. Studying healthcare use among FCs that may participate in future HIV prevention research enables better characterization of health issues affecting these communities. This positively impacts on residents´ health by identifying which health services need to be prioritized for the improvement of health outcomes. Identification of potentially significant health issues that affect FCs, helps understand any unmet needs and additional health care services needed to improve health. Evaluating perceptions of health care and access also helps identify potential factors that affect outcomes in HIV prevention research. Possible factors may include co-morbidities that affect health status, and how residents perceive access to health care in the community (i.e. the impact of perception on individual health-seeking behaviors). In the context of examining healthcare use and access, obstacles that may prevent individuals in the FCs from utilizing health services are also explored. Assessing possible obstacles to using health care resources is important in understanding whether limited access to health services is contributing to increased risk of STIs and HIV acquisition. Understanding how residents of FCs are seeking and using health services will inform how health resources could be improved or tailored to better meet the needs of FCs to improve health outcomes.

We therefore explored the perceived healthcare use, barriers to accessing healthcare, and factors associated with seeking STI symptoms care among fishing communities on Lake Victoria.

## Methods

**Study design:** this study was a mixed methods cross-sectional community-based survey integrated into a larger study called the Community HIV Epidemiology and Socio-behavioral Study (CHESS), which was designed to characterize factors associated with the risk of HIV infection, understand HIV prevalence, HIV incidence, and access to health care in fishing communities.

**Study setting:** this cross-sectional survey was conducted from February 2014 to November 2015 following a household census in three islands (Kiimi, Makusa, and Namisoke) and one mainland (Nakiwogo) fishing community of Lake Victoria, Uganda. The four communities were selected for the survey based on their size following the community household census (two large and two small).

**Participants and eligibility criteria:** a community-size representative random sample of eligible participants (aged 13-49 years at census time and having spent at least 6 months in these communities) was selected to participate in the survey following the household census. All eligible participants in a sampled household were enrolled until the predetermined computer-generated random sample for each community was achieved. Details have been documented elsewhere [6].

Eligible participants were given detailed information about the study through an information sheet read to them in the language they preferred (either English or Luganda). The information discussion was conducted in the presence of a guardian if the participant was a minor and or an impartial witness if he/she was illiterate in the language of the consent. Participants who understood the study information and were willing to take part signed the informed consent document, a copy of which was given to them.

### Data sources and measurement

**Data collection tools:** a semi-structured questionnaire was used to collect quantitative data, while FGD and KII guides were used for qualitative data collection. The data collection tools were developed in English and translated into Luganda the local language.

**Data collection:** training and piloting of the data collection tools were conducted before data collection. The trained research assistants administered face-to-face questionnaire interviews to participants within their homes or workplaces or other convenient locations of their choice, where confidentiality would be maintained. The research assistants collected data on socio-demographic characteristics, self-reported health problems, existing health services, and healthcare-seeking behavior, including whether they sought and received treatment, and where they received treatment (within or outside the community they resided in). Participants were also asked questions about their perceptions of health problems and barriers to addressing them. A health problem was defined as having suffered an illness, condition, or injury during the past 12 months, while individual problems were any illnesses, conditions, or injuries experienced by the participant during the past 12 months before the survey.

Participants for the FGDs were randomly selected and divided into the following four categories (based on gender and age) to facilitate the free flow of discussions and conformity to cultural norms: adolescent girls (13-18 years), adolescent boys (13-18 years), adult women (19-49 years), and adult women and men (19-49 years). Each FGD had 6-12 participants. The KIIs were conducted with purposively selected participants from the community leadership structures (village local council chairpersons, beach management unit leaders, women group leaders, and religious leaders) who were residents and were thought to be informed and knowledgeable about the study communities.

**Study size:** there were approximately 4,232 inhabitants from across all four study communities, of which 2,818 were eligible (aged 13-49 years, had spent at least 6 months in the community before the household census) and 1,469 (52.13%) participants were randomly selected based on the size of each community.

**Statistical methods:** the study aimed to answer the following questions: 1) What are the major health problems in these communities? 2) What factors are associated with seeking care for sexually transmitted infections (STIs) symptoms? 3) What are the main reasons for not seeking care for health problems?

**Quantitative variables and analysis:** the dependent variable was seeking care for STI symptoms (genital ulcers, genital discharge, genital itching, and painful urination) which were coded as “yes” for participants who sought care and “no” for those who did not seek care. Independent variables were selected based on previous literature or biological plausibility and included; participants´ gender, age in completed years at the time of the survey, years of formal education, religious affiliation, tribal affiliation, time spent in the community, marital status at survey time and main occupation.

Frequencies were used to summarize the independent variables, including social demographic characteristics and reported health problems. Bivariable Chi-square tests were used to assess the associations between independent variables and the dependent variable at a 95% significance level. The selection of independent variables for inclusion in the multivariable logistic regression model was based on previous literature, biological plausibility, and a bivariable analysis p-value ≤ 0.2. Model goodness-of-fit was assessed by Akaike´s information criterion (AIC) and Bayesian information criterion (BIC) values. Adjusted odds ratios, P-values, and 95% confidence intervals (CI) were used to report associations. All analyses were done using STATA® version 15 [7], with tables created using ASDOC [8].

**Qualitative analysis:** to obtain comprehensive information on views and experiences of health problems and barriers to addressing them, qualitative data was collected through fourteen FGDs with male and female residents and ten KIIs with religious, village health teams (VHTs), and community leaders.

The Luganda local language FDGs and KIIs were audio-recorded with consent from the participants, transliterated verbatim, and interpreted into English. Data were coded and analyzed manually following a thematic framework approach. Statements were organized into major themes, and representative quotes facilitated conceptual descriptions of the participant responses. Statements were used to convey the pattern of responses only if a theme was expressed by more than one participant, and in more than one group. The contents were translated into English.

**Ethical approval and consent to participate:** the study was approved by the Uganda Virus Research Institute Research Ethics Committee (Federal Wide Assurance (FWA) number 00001354) and the Uganda National Council of Science and Technology (FWA number 00001293). Participants 18 years and above were enrolled after providing written informed consent. Adolescents aged 13-17 years were enrolled after documented emancipated minor consent if they were emancipated minors or assent, with documented consent from their parents or guardians.

## Results

### Quantitative findings

**Participant characteristics:** a total of 1,469 residents participated in the survey. The median (interquartile range) age was 29 (23-35) years, 51.9%, (763/1,469) were females and 60.4%, (888/1,469) had not studied beyond seven years of formal education (Table 1).

**Table 1:** participant characteristics

Characteristic (n = 1,469)	Frequency	Percentage
**Sex**		
Female	763	51.9
Male	706	48.1
**Age (years)**		
Median, IQR	29 (23 - 35)	
13-19	175	11.9
20-34	879	59.8
35-49	415	28.3
**Occupation**		
Fishing and related	563	38.3
Trade/business	175	11.9
Bar/lodge work	177	12.0
Housewife	128	8.7
Others	426	29.0
**Education (years)**		
None-formal	102	6.9
Seven	786	53.5
Thirteen	539	36.7
Over thirteen	42	2.9
**Religion^a^**		
Catholic	577	39.3
Protestant	406	27.6
Muslim	312	21.2
Pentecostal	136	9.3
Others^&^	37	2.5
**Duration of community stay**		
6 - 11 months	134	9.1
1 - 4 years	540	36.8
5 - 10 years	415	28.2
More than 10 years	380	25.9
**Community**		
Mainland	461	31.4
Island	1008	68.6
**Marital status**		
Not married	535	36.4
Married	934	63.6

& traditionalist, no religion, seventh-day Adventist, Isa Messia, Wobushobozi, Orthodox, Amalekite; ^a^: 1 missing religion

**Community health problems and healthcare-seeking:** the most frequently reported major community health problems were: unclean water, poor hygiene, and poor sanitation (WASH)-related (596/1,469, 40.6%) (Table 2).

**Table 2:** major community health problems

Major health problem (n =1,469)	Frequency	Percentage
WASH-related**	596	40.6
Poor health care	329	22.4
HIV/AIDS	203	13.8
Malaria	161	11.0
Cough	69	4.7
STIs	34	2.3
Alcohol and drug abuse	22	1.5
Other	55	3.7

** Waterborne diseases, diarrhea, poor sanitation, lack of toilets and clean water; WASH: water, poor hygiene, and poor sanitation

Poor health care including lack of services, absence of health facilities, lack of skilled health workers, equipment, and drugs were also reported as problems (329/1,469, 22.4%) (Table 2). HIV/AIDS was also reported as one of the most common community health problems (203/1,469 13.8%) (Table 2).

Over 75% of participants reported having suffered from any illness, condition, or injury during the preceding 12 months (76% (1,117/1,469)). A total of 1,752 health issues were described, and participants reported seeking care for 1,429/1,752 (81.6%) of the health issues identified. The most frequently reported health issues were: STI symptoms (genital ulcers, genital discharge, genital itching, and dysuria) (605/1,117, (54.2%)), malaria or febrile illness (418/1,117, (37.4%)), and cough or difficulty breathing (253/1,117, (22.6%)). Most participants who sought care received care (97.9%) (Table 3).

**Table 3:** participants’ health problems and care-seeking

Health problem	Had problems n = 1,117			Gender				Sought care		Received care		Location of care			
STIs symptoms		n	%	F	%	M	%	n	%	n	%	Within	%	Outside	%
	Yes	605	54.2	382	63.1	223	36.9	420	69.4	408	97.1	320	78.4	136	22.6
	No	512	45.8												
**Malaria/febrile illness**															
	Yes	418	37.4	223	53.4	195	46.6	397	95.0	396	99.7	302	76.3	113	28.5
	No	699	62.6												
**Cough/difficult breathing**															
	Yes	253	22.6	143	56.5	110	43.5	224	88.5	221	98.7	192	86.9	35	15.8
	No	864	77.4												
**Diarrhea/stomach pain**															
	Yes	96	8.6	38	39.6	58	60.4	81	89.0	79	97.5	65	82.3	18	22.8
	No	1,021	91.4												
**Dyspareunia**															
	Yes	65	5.8	54	83.1	11	16.9	43	66.2	39	90.7	29	74.4	10	25.6
	No	1052	94.2												
**Pregnancy complications**															
	Yes	16	2.4	16	100	0	0	14	87.5	14	100	8	57.1	7	50.0
	No	585	97.6												
	N/A	516													
**HIV/AIDS**															
	Yes	22	2	11	50	11	50	19	86.4	18	94.7	2	11.1	16	88.9
	No	1,095	98												
**Other problems**															
	Yes	277	24.8	140	50.5	137	49.5	231	83.4	224	97.0	159	71	87	38.8
	No	840	75.2												

**Factors associated with seeking care for STIs:** adolescents,13-19 years at enrolment were less likely to have sought care for STI symptoms than participants 20 years and older (aOR= 0.5 (95% CI 0.3-0.9)). Female participants were more likely to have sought STI treatment (aOR= 1.4 (95% CI 1.0-2.1)). Participants whose main occupation at enrolment was bar or lodge work were twice as likely to have pursued STI symptoms treatment as those who were not working in bars or lodges (aOR= 2.0 (95% CI 1.1-3.6)) (Table 4).

**Table 4:** crude (cOR) and adjusted (aOR) odds ratios of factors associated with seeking STI symptoms treatment

STIs treatment seeking	cOR	95% CI	aOR	95% CI
**Age group (years)**				
20-49	1.0		1.0	
13-19	0.5	0.3-0.9	0.5	0.3-0.9
**Gender**				
Male	1.0		1.0	
Female	1.5	1.1-2.2	1.4	1.0-2.1
**Marital status**				
Unmarried	1.0			
Married	1.4	1.0-2.0	1.4	0.9-2.1
**Education (years)**				
None	1.0			
Seven	0.7	0.3-1.3	0.7	0.4-1.4
8 or more	1.1	0.5-2.2	1.3	0.7-2.7
**Occupation**				
Non-bar/lodge	1.0		1.0	
Bar/lodge	2.0	1.2-3.6	2.0	1.1-3.6

**Reasons for not seeking care:** participants did not seek care for their health problem approximately 18.4% of the time (1,752 medical conditions reported). High care costs, fear (of staff and being stigmatized), and lack of time, were some of the most frequently reported reasons for not seeking care (Table 5).

**Table 5:** reasons for not seeking care for health problems

Reasons for not seeking care	Reason frequency	Percent
Too expensive	201	30.7
Reluctant	97	14.8
Lack of time	93	14.2
Fear (staff and stigma)	89	13.6
Lack of health facilities and personnel	8	1.2
Condition resolved	47	7.2
Treatment is not available	35	5.3
Don’t want treatment	34	5.2
Treatment far away	23	3.5
Others	28	4.3

### Qualitative findings

**Participant characteristics:** a total of 112 participants participated in the qualitative survey, of whom 38 (34%) were adolescents. Adolescents were school dropouts, working in bars, lodges, as loaders, off loaders and casual laborers. Adults mainly worked as fishermen, fishmongers, bar, lodge and restaurant owners.

**Community health problems and healthcare-seeking:** water, poor hygiene, and poor sanitation (WASH) problems were also noted during FGDs and KIIs; *“I do not go to latrines instead go to the bush because every time I would go to the toilet, I would get candidiasis, treat, and cure but would get it again until when I stopped going to the latrine. I did not get it again. Some are already full; they need to be emptied and when you enter you see as if you are going to fall inside. Some people cannot afford to pay for the toilet fees”* KI male.

*“There is only one source of clean water (tap) that is not enough. People wait for many hours, and even then, it is often broken down/spoilt. This has often forced people to get water directly from the lake”* FGD female.

A lack of services; including the absence of health facilities, lack of skilled health workers, equipment, and drugs were also problems in these communities from the FDGs and KIIs; *“Health workers at times take 3 months to provide HIV Counseling and Testing (HCT) in the form of outreach, when I have gotten a new woman today, by the time I wait for the three months for the health worker to come, then I will miss out or end up having sex without testing for HIV”* FGD women and men.

Lack of health services was also one of the reasons for not seeking care, as indicated during the FDGs and KIIs; *"only private clinics are available yet very expensive. Sometimes we cannot afford the bills and transport to the mainland is a problem. When one´s illness worsens at night, it is hard to transport him/her because there is no transport……. at times community members have to contribute to transport the sick person”* FGD female.

*"I do not have money to transport myself to go for treatment, so I end up missing getting drugs (ARVs), if you have not taken the treatment for 3 days you really fall sick. This affects patient adherence. We at times encouraged people in HIV care to contribute towards transport, hire a boat, and go at once but this is only to those who opened up to some of us, if they fail, we have to get from our own pockets to help our clients”* KI female.

## Discussion

This study highlights major health problems affecting these fishing communities, with STIs being the most frequently reported individual health problems, a key driver of the HIV epidemic. Participants reported WASH-related problems and poor health care as the most frequent community health issues. Fishing communities are hard to reach due to maritime transport challenges with limited access to social services. These situations may predispose to WASH-related health problems due to challenges accessing health care, information, and education. Most households in these communities have no latrines [9,10], partly due to the sandy nature of the soils, making it expensive to construct latrines. Such households resort to bushes, the lake, or “buvera” (polyethylene bags) as avenues of convenience, which precipitates WASH-related health problems [9]. Lower levels of formal education, with the majority having not gone beyond seven years, may make it harder to comprehend WASH disease prevention-related information.

Water, poor hygiene, and poor sanitation (WASH)-related health issues have been associated with reproductive tract infections in similar settings, with the latter being more frequent among those practicing open defecation compared to those using pit latrines and those using water after visiting the latrine compared to those that do not use water [11-13].

Poor healthcare was reported frequently as a community problem, often specified as a lack of/or poor health services, lack of health facilities, facilities being far away from fishing communities, and lack of health supplies including drugs. This has also been noted in other Ugandan settings [14,15]. Healthcare in some fishing communities is infiltrated by unqualified or unskilled personnel, as the few skilled workers find it hard to work in these communities due to maritime travel challenges. As most of these communities are islands, it is challenging to seek care from a neighboring island community for a service lacking in the participant´s community considering the high cost of hiring a boat, and engine, paying the coxswain and fuel.

STI symptoms were among the most frequently reported health issues. This finding may have significant implications for treating STIs in these communities and specifically may impact rates of HIV transmission as STIs have been shown to increase the likelihood of HIV transmission [16]. These communities have a high burden of HIV [17,18] and STIs have been associated with HIV [2]. Members of these fishing communities engage in unprotected sexual relationships due to inadequate access and low condom use [19-21], with the perception that HIV is less life-threatening compared to the daily challenges of lack of shelter, food, or clothing [22]. This altered risk perception and a belief that HIV acquisition is not within an individual´s control makes FCs members engage in unprotected transactional sexual relationships for survival increasing the risk of acquiring STIs [23,24].

Female participants were more likely to have sought STI treatment than males. This is probably due to better health-seeking behavior among females than males [25,26]. It could also be that STIs affect more females than males due to their social, and economic-cultural vulnerability, limited ability to negotiate safer sex, and anatomical susceptibility [21,26]. However, in other settings, females were found to seek care less frequently than males [27], possibly due to socio-cultural and formal education differences between communities. Male gender norms of self-sufficiency, resilience, and being embarrassed might have led to many men in these communities to not seek care for health problems [27]. Higher formal education has been linked to better healthcare-seeking and receipt [28,29]. Although this study found that participants with at least eight years of formal education were more likely to seek STI treatment, these findings were not statistically significant.

Adolescents were less likely to have sought treatment for STI symptoms. Although we did not survey the health facilities in these communities, it´s possible that the services were inadequate and may have lacked adolescent-friendly services that have been documented to improve access to care among this age group [30,31]. A healthcare environment without adolescent-friendly services might have made it harder to seek treatment for STIs, probably due to challenges reporting STI symptoms to older care providers who might have demeaned them or even disclosed their health problems to guardians. Adolescents in these FCs could also have lacked knowledge of STIs which has been linked to not seeking care [31]. Adults might also have had multiple previous STIs health care seeking experiences, with prior knowledge of where and how to seek care for these symptoms compared to adolescents. We however did not ascertain prior knowledge of where to access care for the different health problems among participants. This highlights the need for further research on understanding adolescents´ access to and use of adolescent-friendly services for STIs and HIV prevention in these FCs.

Participants mainly working in bars and or lodges were more likely to seek treatment for STI symptoms. This occupation group has been previously found to have a higher burden of HIV [16]. Bar or lodge workers are more likely to be engaged in sex work and transactional sex, as some of them do not earn a salary but are rather paid based on the number of customers they attract to the bar/lodge. This makes them vulnerable to sex work and transactional sex for survival, increasing their frequency of having STIs and seeking treatment for them.

HIV/AIDS-related health problems identified in this study were relatively fewer compared to the HIV burden in these communities perhaps due to uptake in antiretroviral therapy [32]. It may be that participants feared being stigmatized for reporting a history of HIV/AIDS-related symptoms. It is also likely that those who got HIV/AIDS-related symptoms left the communities earlier for care and to avoid being stigmatized, as the majority of people living in fishing communities are mobile, having left their permanent homes on the mainland to earn a living in these communities [1]. It´s also possible that the participants who agreed to participate in the study were more likely to seek care or have fewer HIV-AIDS-related symptoms for other reasons.

For most of the reported health problems, participants sought treatment most often from within their communities, meaning they did not travel outside of their community to seek care. Maritime challenges and the costs of seeking care outside participants´ and communities may have caused many to seek care from within the community [33]. It seems that for HIV/AIDS care, treatment was mainly sought outside participant's communities as these communities had limited HIV/AIDS treatment services with most community members seeking care from mainland facilities. They might also have sought care outside to avoid being stigmatized within their communities.

The main reason for not seeking care was that treatment was too expensive, so focusing on making services more affordable would perhaps improve treatment uptake for these fishing communities. A limitation of this study was the use of self-reports with no medical records to compare with these findings. Recall bias was also a challenge though minimized using a shorter duration of recall (12 months). The study findings, despite the limitations are generalizable to fishing communities on Lake Victoria.

## Conclusion

Participants reported WASH-related issues as major community health problems. Healthcare use in these communities was characterized by seeking care within participants´ and communities, with adolescents being less likely to have sought care for STI symptoms. Barriers to health care seeking were mainly the high cost of care.

### What is known about this topic

- *Fishing communities on Lake Victoria are hard to reach;*
- *Fishing communities´ members have a high HIV burden.*

### What this study adds

- *Water, poor hygiene, and poor sanitation (WASH)-related problems are still a challenge in fishing communities;*
- *There is perceived poor health care with high costs;*
- *Adolescents are less likely to seek STI care in these communities.*
